# Transpalpebral extrusion of solid silicone buckle

**DOI:** 10.4103/0974-620X.53040

**Published:** 2009

**Authors:** Abadan Amitava Khan

**Affiliations:** Institute of Ophthalmology, JN Medical College, AMU, Aligarh, India

**Keywords:** Explants, reattachment surgery, transpalpebral extrusion

## Abstract

Explants used in retinal reattachment surgery occasionally extrude. Cheese-wiring of the suture through the sclera consequent to raised intraocular pressure allows the buckle to loosen and/or unfold. Subsequent infection, often with Staphylococcus albus, accelerates the process of extrusion. Commonly, such explants are of silicone sponge. The reported case is unusual in that the extrusion occurred through the upper lid, and involved a solid silicone explant.

## Introduction

In retinal reattachment surgery, extrusion of silicone buckling material, whether sponge or solid silicone, is a known complication. The reported incidence varies from 0.5% (in primary surgery) to 10% (in revision surgery).[1] We report a case of solid silicone explant extrusion occurring a decade after buckling surgery. This case is of considerable interest since the solid silicone band extruded through the upper lid; an extremely rare occurrence. Transpalpebral extrusion has rarely been reported and largely with silicone sponge.[2-4]

## Case Report

A 36-year-old male presented with a sore right eye of six weeks duration. Before six weeks he had experienced pain with a foreign body sensation on the under surface of the upper lid, and after three days he noticed a yellowish discharge. A week later he perceived a diminution of vision in the right eye, associated with photophobia, and a whitish lesion on the black portion. His past history revealed that he had undergone uncomplicated intracapsular cataract extraction in the right eye 18 years earlier, and in the left eye 15 years ago; for developmental cataracts, for which no etiology could be ascertained. Also, 10 years ago he underwent an encirclage of the right eye for aphakic rhegmatogenous retinal detachment, with a solid silicone equatorial band. He had regained useful vision, but he had not maintained follow-up visits.

**Figure 1:**
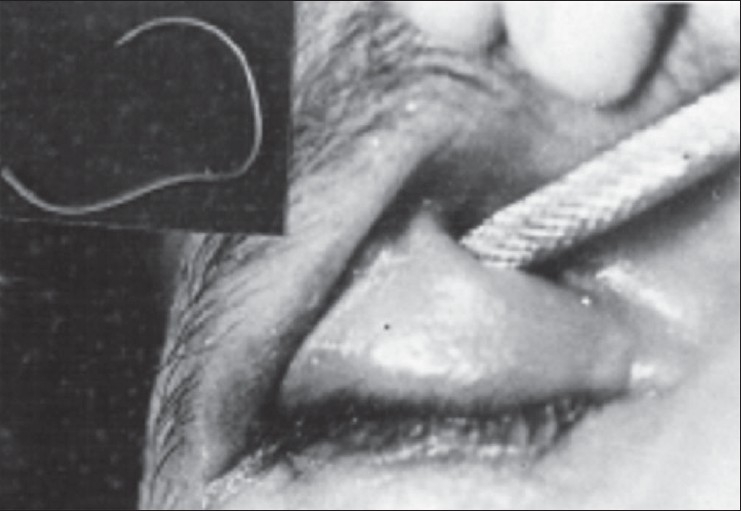
The transpalpebral defect is highlighted with a metal probe. Inset shows the solid silicone band, which extruded, and was surgically extracted

Examination revealed visual acuity of hand movement in the right eye; and 6/9 with +9.5 DS/–2 DC X 180°, in the left eye. The right upper lid was ptotic, swollen, indurated, and mildly tender, with a horizontally oval fistula in the superonasal aspect, approximately 1.5 X 1 cm in size with well rounded margins [Figure 1], and purulent discharge oozing out. On cleaning the area, a free end of a silicone band could be identified. Examination of the right eye revealed an oval hypopyon corneal ulcer, on the inferior one-third of the cornea, with dirty dry looking sloughing material, and purulent discharge on the lashes. The upper fornix had been obliterated, with areas of symblepharon. The central fundus appeared normal on indirect ophthalmoscopy; the periphery could not be evaluated. The left eye was aphakic and otherwise unremarkable, except for few areas of lattice in the superotemporal quadrant. There was marked limitation of ductions and versions of the right eye, more so for the vertical movements. Gram stain and cultures identified the organism as Staphylococcus albus. The patient underwent silicone band removal [Figure 1, inset], wound toilet with betadine 5%, and was placed on topical ciprofloxacin (hourly during day; and two hourly at night, for the first 48 hours; and later tapered), and systemic cefotaxime (1 gram intravenous twice daily for three days, then switched to intramuscularly twice a day for four days), along with atropinization. There was no evidence suggestive of rectus muscle disinsertion on forced duction and generation testing. After two weeks, substantial healing of the corneal ulcer and narrowing of the sinus opening was observed. Subsequently, the patient was lost to follow-up.

## Discussion

Explants of various materials have been reported to have extruded. The initial event often is cheese-wiring of the suture through the sclera, permitting implant migration, one end of which may then erode the conjunctiva. Subsequent infection often compounds this process, frequently hastening implant migration due to necrosis of the overlying conjunctiva. The initial event usually also involves a breach of the conjunctiva, either by a sharp corner of one end of the buckle (made loose due to breakage of suture), or through external injury. On occasion, the event is triggered presumably by some allergic reaction to the implant material.[1] Such extrusions may occur after varying lengths of time subsequent to retinal reattachment surgery, ranging from two months to 15 months.[5] MIRA gel scleral buckle extrusion through the lower lid has been reported 10 years after scleral buckling.[6] Rolland-Pollares,[7] in a study (1999) of 757 patients, found that removal of explant was necessitated in 1.3%. Silicone sponge was removed in three of 32 patients (9%), shortly after surgery. Silicone rubber was removed in two of 360 patients (0.6%); on an average of one year postoperatively. MIRA gel required removal in five of 386 patients (1.3%) after long-term follow-up of almost 7–10 years. Silcone sponge and rubber showed the presence of infection with positive cultures, whereas MIRAgel cultures were positive in 20% cases. The large majority of extrusions are associated with infection: the most common organism being Staphylococcus albus, followed by Staphylococcus aureus, and Proteus.[2] Both MIRA gel and silicone sponge buckles are extremely soft and erosion of the ocular tissues alone is an inadequate explanation to the process of extrusion, perhaps infection or allergy may more frequently compound the process.

Our case is unique in that, a solid silicone buckle extruded spontaneously through a defect in the upper lid after almost nine years, a finding not hitherto reported. Maguire reported two cases, wherein solid silicone buckle extruded by cheese-wiring through the recti muscles.[8] Cheese-wiring implies cutting through muscle tissue with subsequent reunion, in contrast to disinsertion of the muscle insertion, wherein an obvious paralysis of the concerned muscle would be evident. Five similar cases were reported by Lanigan, two of which had ocular motility problems.[9]

In our case, it is likely that the initial event may have been the sutures cutting through the sclera, once the intraocular pressure was restored or exceeded normal levels postoperatively, thereby loosening the implant. Infection further enhanced the disintegration of sutures. This permitted the silicone buckle to migrate anteriorly. It is probable that the patient developed superior symblepharon prior to buckle migration, contributing to lid immobility. Hence, the end of the buckle would have directly abutted on the undersurface of the upper lid. More conceivably the buckle migrated through the superior orbital tissue and hence through the eyelid. Over time it formed a sinus track, superior to the upper border of the upper tarsal. It is difficult to explain the occurrence of the corneal ulcer in the inferior aspect of the cornea. Presumably some breach of the conjunctiva may have occurred at some time and infective discharge, associated with the chronic conjunctivitis which set in, predisposed to an ulcer following some subclinical trauma of the corneal epithelium. Undoubtedly, in this case, the late presentation and poor follow-up on the part of the patient was responsible for this unusual presentation. This serves to highlight the importance of educating the patient to seek prompt consultation when there is pain and discharge, even many years after successful retinal reattachment surgery. In such instances, orbital imaging should form a vital aspect of work up since it will help to reveal the current position of the buckle and associated muscle complications.
